# Normative based beliefs as a basis for perceived changes in personality traits across the lifespan

**DOI:** 10.1371/journal.pone.0264036

**Published:** 2022-02-17

**Authors:** Joanna Gutral, Marzena Cypryańska, John B. Nezlek

**Affiliations:** 1 Department of Psychology, SWPS University of Social Sciences and Humanities, Warsaw, Poland; 2 Institute of Psychology, Center for Climate Action and Social Transformations, SWPS University of Social Sciences and Humanities, Warsaw, Poland; 3 Department of Psychological Sciences, College of William and Mary, Williamsburg, Virginia, United States of America; University of Padova, ITALY

## Abstract

This article presents a new framework for understanding how people think personality changes across the life span. In two studies we examined the correspondence among how people thought their personalities would change, how people in general change, and changes found in a meta-analysis of changes in personality. We conceptualized and measured personality in terms of the Big Five model (FFM). In Study 1 participants rated either how they had changed from the past to the present or how they would change from the present to the future. We found that for openness to experience and social vitality participants thought these traits had increased from the past to the present, whereas participants did not think they would change from the present to the future. In contrast, participants thought that conscientiousness, agreeableness, and emotional stability would increase from the present to the future, although they did not report changes in most of these traits from the past to the present. The changes that occurred in Study 1 correspond to changes of personality found in previous research. In Study 2 participants rated themselves and other people on the FFM traits for each of nine intervals representing the lifespan. We found that people perceived changes in themselves to be similar to the changes found in meta-analyses, and perceptions of change in the self-corresponded to perception of changes for others. We believe these results can be explained by recognizing that people share normative based beliefs about how people change at certain age. Nevertheless, we also found that people perceived themselves as better than others, i.e., relatively greater increases in some positive traits and relatively smaller decreases in some negative traits, being first among equals. We discuss possible explanations for this phenomenon, which according to our knowledge, has not been discussed in this context previously.

## Introduction

In this paper we propose and test a new model explaining people’s expectations about how their personalities will change over their lives. The model assumes that peoples’ expectations about how their personalities will change over the course of their lives are informed by normative-based beliefs about how people in general change over the lifespan. When thinking about what they will be like in the future, people base their beliefs on the experiences of others, at least in part.

Basing judgments about the self on the experiences of others introduces the possibility of tension between the need to belong, to be part of a social group, and the need to be an individual with a distinct identity [1]. Although people like to see themselves as unique, differentiated individuals [2,3], they also tend to assimilate to the norms of their groups [4]. There is also the issue of people’s needs to maintain positive self-evaluations, including self-enhancement, people’s desire to maximize the positivity of their self-views [5], which can manifest itself in a tendency to see one’s self as better than others [6]. Consequently, people may want to see themselves simultaneously as similar to others and better than others.

In terms of people’s beliefs about how they will change over the life course, these various influences can be manifested as tensions between people’s beliefs that they are maturing and will age in unique ways (perhaps more favorably) and beliefs that they mature and will age as everyone else does. We believe that one way to balance the need to belong and the need to be different from and better than others is to see oneself as conforming to the desirable norms but in a more privileged way than other people. The existence of such a possibility was confirmed by Codol [7] who labelled this tendency as the “superior conformity of the self” or the “primus inter pares” (PIP, first among equals) effect. The present model incorporated the PIP as an explanation for perceived changes in personality across the life span, something that we believe has not been done previously.

Generally, we thought that people would expect that their personalities would change over the lifespan in ways that were similar to how they thought other people would change, i.e., a normative pattern of personality development. Nevertheless, people would simultaneously expect that changes in their own personalities would be more desirable than changes in others. For example, for a positive characteristic such as vitality, although people may believe that their vitality will decrease over time, people may think that the decrease they experience will be less or later than the decrease the typical person experiences. In contrast, for a positive characteristic such as emotional stability that is associated with a mature behavior [8], although people will expect that others will become more mature and stable over time, people will expect that they will improve over time more or faster than others will improve.

### Personality as a context for examining perceived changes across the lifespan

We examined how people think people change across the lifespan in terms of personality because there is an established body of research about such perceptions and about actual changes in personality. This provided a basis to examine the correspondence between perceived changes and actual changes. Although personality traits are often treated as stable individual differences, studies of personality across the life span indicate that people change and that such changes are somewhat normative [9]. Aside from whether people in fact change, there are also questions about people’s perceptions of such changes. Research on such perceptions fall into two broad categories, research about how people think they themselves will change [10] and research on how people think other people will change [8].

Despite the conceptual overlap among these three bodies of research, changes in personality and perceived changes in self and in others all concern changes in personality over time, we are unaware of any study that has examined the three of these topics together. Moreover, the ability to examine relationships between perceived changes in the self and in others and relationships between these perceptions and normative changes should provide a basis to further our understanding of the processes underlying people’s beliefs about how personality changes across the lifespan.

To address such issues, participants in the present study described what they thought they would be like at different points in their lives. They also described what they thought people in general would be like at these same points. Descriptions were made in terms of the traits of the Five Factor Model (FFM), and age intervals were chosen to correspond to the intervals used by Roberts et al. in their meta-analysis of changes in personality across the lifespan [9]. This combination of descriptions and intervals allowed us to examine the correspondence between normative changes (i.e., changes from the meta-analysis) and how people thought they would change and the correspondence between normative changes and how people thought other people would change.

### How does personality change across the lifespan?

The results of a meta-analysis of 92 longitudinal studies found that with age, people become more socially adapted, agreeable, conscientious, and emotionally stable [9]. Consistent with this, cross-sectional research has found that people in middle-age are more agreeable and conscientious than younger individuals [11]. In contrast, for traits that are associated with vitality and exploration (e.g., extraversion and openness to new experience) decreases over time have been found [12]. Additionally, analyses at the mean-level changes over the lifespan have found that people demonstrate an increase for self-confidence, self-control and emotional stability over the lifespan mostly in young adulthood [13].

Although research has found that personality changes are larger between the ages 20 and 40 than they are between other ages, change also occurs in adolescence, young adulthood, and in older ages [9]. In other words, it appears that personality changes across the lifespan, defined for present purposes as 10–80 years old. Personality change in early childhood is a distinct topic that is not considered in the present paper.

In general, the existing research has found that that conscientiousness and agreeableness increase with age, whereas extraversion and openness decrease with age [9,12]. Although not as consistent as these relationships, research has found that neuroticism tends to decrease with age [14]. These changes in personality over the life span represent what has been referred to the “maturity principle,” increases in the attributes associated with positive adaptation and in the attributes associated with the fulfillment of adult roles during adulthood [8]. These changes can be also interpreted in terms of Digman’s [15] alpha and beta factors, increases in traits related to community coexistence and social interest (alpha), and decreases in traits related to agency and active realization of life goals (beta).

Moreover, people seem to be aware of these changes and their perceptions of the direction of normative adult personality changes over the lifespan tend to be reasonably accurate [16]. Research on the effects of age-graded roles in forming expectations for changes in the Big Five personality traits has found widely shared the expectations for behavior at different ages or in age-graded roles, and these expectations may represent widely shared cultural norms [8]. Although these expectations correspond to the changes that have been found in studies of actual change [9], it is not clear if people use widely shared expectations about personality traits changes when they form expectations about change over the lifespan in their own personalities. We are not aware of a study that has measured perception of changes in the self over the lifespan and how these changes correspond to perception of changes of other people.

### Perception of the self over the life course

Previous research has not examined if people’s perceptions of how their personalities change across the lifespan correspond to the changes found in studies of personality development, although there are reasons to believe they do. First, it is clear that people see themselves as changing across the lifespan in terms of personality traits [10,17], competence [18], attractiveness [19], and well-being [20]. Second, the pattern of these perceived changes seems to reflect beliefs about patterns of maturation and aging. Specifically, people seem to share the general belief that up to a certain point in life, various qualities increase and improve, and then, from a certain point in late adulthood, various skills and characteristics decline [11].

For example, research on the perception of personal progress and decline over the course of adult life has found expectations of increases in well-being over the lifespan, up to the late 60s and decreases in well-being after that [20]. Similarly, Heckhausen and Krueger [17] found that people expected declines in many desirable characteristics (except wisdom) in late adulthood. Studies on subjective beliefs of personality development [10] have found that people perceive moderate and continuous changes in their personality traits during the adult life span. The pattern of these perceived changes reflected expectations of more losses than gains in late adulthood, and it was characterized by early adulthood exploration, productivity in middle adulthood, and finally comfortableness in later adulthood.

### Self-enhancement motive in perception of personal changes over the life course

In parallel to the research on perceived changes in personality and well-being, there are bodies of research on temporal comparison [21] that concern how people see themselves in comparison to their past selves (what they were like in the past) or their future selves (how they expect to be in the future) [22,23]. This research has focused on people’s perceptions of whether they have improved over time and on their perceptions of whether they expect to improve over time, and the changes usually referred to specific time intervals (e.g., 6 months, 2 years, 10 years).

This research has examined perceived changes in personality traits [22], and other traits that have been assumed to be desirable (e.g. polite, creative) and undesirable (e.g. jealous, lazy) [23,24]. It has been found that people tend to believe they have improved from the past to the present [22,24]. Some studies have also found that people expect that they will improve from what they are in the present to the future, with future defined as both near-term (e.g., 6 months) and longer‑term (e.g., 2 years) [19,23].

Perhaps the most widely accepted explanation for these positivity effects in temporal comparison is based on the self-enhancement motive. According to this explanation, people devalue their past selves and idealize their future selves to maintain or enhance their current self-regard or self-evaluation [24]. In this paper we consider the influence of self-enhancement motives on people’s temporal ratings within the context of social comparison, defined as how people see their personal changes in personality over the life course in comparison to how they perceive the changes in the personalities of people in general. Assuming that the perceived personality changes are informed by normative-based beliefs about how people change over the lifespan, the pattern of perceived personal changes over the life course does not need to reflect improvement over the lifespan. It is possible that for some traits, such as extraversion or openness, people can expect declines over time.

Nevertheless, even when people believe they will decline over time as other people do, they can still self-enhance by expecting to be better than others by believing that they will decline less than other people. An informative context for considering such a possibility is Codol’s “superior conformity of the self” phenomenon [7]. According to Codol, people tend to present themselves as conforming more to social norms than other members of their societies. This tendency results from a conflict between two tendencies that are both complementary and contradictory. On one hand, people’s need for social conformity leads to searching for social similarity and standardization (which is why we expect that people will think their personalities will change in ways that are similar to how other people will change), On the other hand, people also seek individualization and social differentiation. The best way for people to reconcile these two tendencies is to be better than other people in terms of what is normative and socially desirable.

We believe that people do not expect increases in all positive characteristics nor decreases in all negative characteristics. People expect to change as other people do, increasing in terms of some traits and decreasing in terms of other traits. At the same time, people can self-enhance by expecting that the changes they experience will be more favorable than the changes others experience. Note that the previous research on temporal comparison did not analyze evaluation on particular personality dimension; rather, it focused on aggregated data for a set of traits instead [19,22,23]. Furthermore, this research focused on temporal comparison that referred to limited time intervals (e.g., 10 years ago or in 10 years) instead of the whole lifespan. These conditions did not provide a basis to examine the types of relationships with which we are concerned.

## The present study

In the present paper we examine a new, preliminary model that is intended to explain people’s expectations of how their personalities change over their lives. We assumed that:

1. Peoples’ expectations of how they will change over the course of their lives are informed by normative based beliefs about how people change over the lifespan.
2. Perceived changes in personality can reflect either systematic improvement or decline over the life course. For example, agreeableness and conscientiousness tend to increase with age whereas extraversion and openness tend to decrease [14].
3. As suggested by Codol [7], although people’s expectations are influenced by normative beliefs (#1), people will tend to perceive themselves to be superior relative to others that can represent a desire to self-enhance (PIP effect). For example, although people will expect declines for some positive characteristics, expected declines for the self will be smaller than the declines expected for others.

We tested three primary hypotheses.

1. People’s beliefs about how their personalities change over the lifespan will correspond to how they think people in general change over the lifespan (normative beliefs).
2. People’s beliefs about changes in their personalities and their beliefs about changes of people in general will correspond to the changes in personality over the lifespan that have been reported in longitudinal studies.
3.Although people’s beliefs about how their personalities change over the lifespan will correspond to how they think people in general change over the lifespan, expectations about changes in the self will be consistent with Codol’s PIP effect and self-enhancement motive.
3a. Participants will expect to experience increase over the life course in agreeableness, conscientiousness, and emotional stability, and they will expect declines in extraversion and openness to new experience.
3b. For traits for which participants expect to increase over the life course, they will expect that they will increase more than others, whereas for traits for which participants expect to decline over the life course, they will expect to decline less than other people do.

These hypotheses were examined in two studies. Study 1 examined the correspondence between people’s beliefs about changes in their personalities and changes that have been reported in the research on personality changes over the lifespan [8]. Study 2 examined the correspondence between people’s beliefs about changes in their personalities and people’s expectations of how others would change (normative-based beliefs) and the correspondence between both of these expectations and changes that have been found in changes in personality over lifespan.

### Study 1

Study 1 examined how people think their personalities change over time, and we focused on a simple distinction between perceptions of the past vs. the future. Participants (aged 18–40) described themselves as they are now, as they were in the past (10 years ago), and as they think they will be in the future (in 10 years). Participants made these rating using the Ten Item Personality Inventory [25]. These data allowed us to determine if reported changes from the past to the present and predicted changes from the present to the future corresponded to the general pattern of personality changes that have been reported in the previous research [8].

#### Method

Participants were 140 college students (113 women, aged 18 to 40 years old, *M*_age_ = 24.91, *SD* = 5.48), who participated in exchange for course credit. They gave their consent to participate in a study examining “The perception of the self at different time perspectives.” They were informed that participation in the study was anonymous and that they could withdraw from participation in the study at any time. Participants had the right to refuse to answer any question.

All participants rated themselves twice in terms of the 10 traits measured by the TIPI. One group of participants (present-past condition, *n* = 74) rated the extent to which the traits of the TIPI described them currently (*I see myself as…*), and the extent to which the traits characterized them ten years ago (*“10 years ago I perceived myself as a…*). In the other group (present-future condition, *n* = 66), participants rated the extent to which the traits of the TIPI described them currently and the extent to which the traits would describe them in 10 years (*“In 10 years I will perceive myself as a…*). All ratings were made using a 7-point scale with endpoints labelled 1 (*disagree strongly*) and 7 (*agree strongly*).

Data files are available at the following OSF site: https://osf.io/7bw35/?view_only=95406e750bec441a83cee94e65694a03.

#### Results

The data were analyzed with two sets of repeated-measures t-tests. One set analyzed the responses of participants in the present-past condition. Ratings of each personality trait for the present were compared to ratings in the past for the same trait. The second set analyzed the responses of participants in the present-future condition. Ratings of each personality trait for the present were compared to ratings of the future for the same trait.

The results of these analyses are presented on Table 1. Note that half of the items on the TIPI are reversed, e.g., extraversion is measured using “reserved, quiet.” We did not reverse score these items prior to analysis. As expected, participants thought they were more open and extraverted now than they were in the past, but they did not expect that openness and extraversion would change from the present to the future. This pattern of results corresponds to the results of a meta-analysis [9] that found that openness increases until adolescence and then remains the same until old age, and social vitality (a facet of extraversion) increases from childhood to adolescent and decreases late in life. This interpretation reflects the fact that the participants in the Study 1 were young adults, aged 18–40, and the time interval for the past and future ratings equaled 10 years.

**Table 1:** Means (and standard deviations) of the difference scores between the ratings for the present minus the ratings for the past/future for five personality dimensions, with the significance test of the difference from 0 (one-sample t-test).

		Present–Past	Present–Future
*Agreeableness*			
	Sympathetic, warm	0.05 (1.22)	-0.36 (0.99)**
	Critical, quarrelsome	0.15 (2.02)	0.42 (1.60)*
*Conscientiousness*				
	Dependable, self-disciplined	0.24 (1.70)	-1.06 (1.25)***
	Disorganized, careless	-0.66 (1.62)**	0.68 (1.52)**
*Emotional stability*			
	Calm, emotionally stable	0.10 (1.73)	-0.88 (1.35)***
	Anxious, easily upset	-0.37 (1.73)	0.82 (1.58)***
*Openness to experience*			
	Open to new experiences	0.64 (1.76)**	-0.26 (1.13)
	Conventional, uncreative	-0.68 (1.48)***	0.14 (1.19)
*Extraversion*			
	Extraverted, enthusiastic	0.47 (1.79)*	-0.18 (0.93)
	Reserved, quiet	-0.64 (1.96)**	-0.29 (1.37)

*** *p* < .001.

** *p* < .01.

* *p<* .05.

Also, the pattern of ratings for emotional stability, conscientiousness, and agreeableness corresponds reasonably well to the results of a meta-analysis [9]. The results of this meta-analysis indicate that emotional stability increases throughout the lifespan until leveling off in the 40s and 50s, conscientiousness does not change from childhood to adolescence, but it increases markedly from young adulthood to midlife. Agreeableness remains relatively constant through the life span until older adulthood (50+) after which time it tends to increase. Although participants did not report changes in emotional stability and agreeableness from the past to the present, they consistently thought that emotional stability, conscientiousness, and agreeableness would increase from the present to the future.

These results support our contention people think their personalities will change and have changed in ways that correspond to changes in personality across the life course that have been reported in the previous research [9,11].

Additionally, we conducted analyses that corresponded to the previous research on temporal comparison [23,24]. First, we calculated the mean of the ratings for all positive traits and separately for all negative traits (the means are presented in Table 2). Next using the series of t tests, we test whether the ratings for the present self were better (higher for positive traits and lower for negative traits) than the ratings for the past self, and whether the ratings for the future self were even better than the ratings for the present self. Consistent with the previous research on temporal comparison, these analyses found a tendency to view the self to be better now than in the past, and the expectation to be even better in the future.

**Table 2:** Means (and standard deviations) for the past, present and future ratings of the positive and negative TIPI traits with the significance test of the difference between past and presents ratings, and present and future ratings (paired sample t-test).

Traits	Past	Present	t	Present	Future	t
Positive	4.83 (0.85)	5.12 (0.63)	2.90**	5.22 (0.79)	5.77 (0.70)	-7.26***
Negative	3.52 (1.00)	3.08 (0.66)	-3.86***	2.98 (0.84)	2.63 (0.90)	3.76***

*** *p* < .001.

** *p* < .01.

### Study 2

Study 2 examined the correspondence between how people think their personalities will change (and have changed) and their beliefs about how people in general change. We also examined the correspondence between changes that have been found in research on changes in personality across the lifespan and how people think they will change (and have changed) and their beliefs about how people in general change. Using the FFM as a context, participants provided two ratings for each of nine age intervals representing the life span. The nine intervals corresponded to the intervals that Roberts et al. used as a framework for their meta-analysis. One set of ratings concerned what they thought they were like in the past and what they thought they would be like in the future. A second set of ratings concerned what they thought people in general were like during different parts of their lives.

#### Method

*Participants*. Participants were 70 students who participated in partial fulfillment of course requirements (52 women, *M*_age_ = 24.51, *SD* = 4.73, aged 19 to 39). Participants provided informed consent, responses were collected anonymously, and participants were told that they could discontinue participation at any time and still receive course credit.

*Measures*. We conceptualized personality in terms of the FFM: emotional stability, extraversion, openness to experience, agreeableness, and conscientiousness [26]. Participants were given definitions of each trait, and we measured how they perceived changes in personality over the lifespan by asking them “To what extent does each trait characterize you in the following periods of life?” Participants answered this question for nine age intervals: up to 10 years old, 10–18 years old, 18–24, 24–30, 30–40, 40–50, 50–60, 60–70, 70+. Participants also described how they thought people in general changed by answering the question “To what extent does each trait characterize people in general in the following periods of life?” They answered this question for the same nine intervals that were used for self-descriptions.

The nine intervals we used correspond to the nine intervals used by Roberts et al. [9] in their meta-analysis of changes of personality over the lifespan, with two modifications to accommodate the years typically spent in college in the country in which the study was conducted. The third interval we used was 18–24, the years most students spend in college (Roberts et al. used 18–22), and the fourth interval we used was 24–30 (Roberts et al. used 22–30). Using 7-point scales with points labeled 0 (*not at all*), 3 (*moderately*), and 6 (*extremely so*), participants indicated how characteristic each trait was for each time interval.

To maximize the likelihood that participants were using the same bases for their answers, we provided a definition of each trait. Openness was defined as “how much someone seeks and is comfortable with novel or new experiences and ideas,” emotional stability was defined as “how much someone is emotionally balanced, calm, able to manage stress effectively,” agreeableness was defined as “how interpersonally agreeable is someone, how much someone avoids interpersonal conflict and seeks interpersonal harmony,” conscientiousness was defined as “how orderly, punctual, reliable and concerned with details someone is.”

To correspond to definitions used in previous research on changes in personality across the life span [9,27,28], we measured extraversion in terms of social vitality and social dominance. Social vitality is defined in terms of traits such as sociability, positive affect, gregariousness and high energy level, whereas social dominance is defined in terms of dominance, independence, and self-confidence in social contexts. Previous longitudinal studies on changes of personality over the lifespan suggested that those two facets present different development patterns. Social dominance tends to increase with age time, whereas social vitality tends to decrease [27,29]. Social vitality was defined as “how much someone is gregarious, socially active, energetic, and enthusiastic,” and social dominance was defined as “how independent, self-confident and assertive someone is.” All data files are available via the OSF cite mentioned previously.

#### Results

*Overview of analyses*. We conceptualized these data as a multilevel data structure in which ratings for intervals were treated as nested within participants. We conducted separate analyses for each trait and separate analyses for self and other ratings. Unless noted otherwise, all slopes were modeled as randomly varying. The analyses were done using the program HLM [30], and the analyses followed guidelines suggested by Nezlek [31]. When interpreting the results of these analyses, it is important to keep in mind that these coefficients are unstandardized. A coefficient represents the expected change in an outcome associated with an increase of 1.0 in a predictor.

*Trends across time*. The first analyses examined trends across time in how characteristic participants thought each trait was for themselves and for people in general. In these analyses ratings (*i*) were nested within *j* persons, and ratings were regressed on two predictors representing the decomposition of the changes across time into two orthogonal components: a linear trend (-4, -3, -2, -1, 0, 1, 2, 3, 4) and a quadratic trend (28, 7, -8, -17, -20, -17, -8, 7, 28). Predictors were entered uncentered. The model is below, and the results of these analyses for ratings of the self and of others are summarized in Table 3. The hypotheses of interest are tested at level-2 via the significance of the γ_10_ and γ_20_ coefficients.


$$\begin{array}{ll} Within‐person & y_{ij}=\beta_{0j}+\beta_{1j}\;*\;linear+\beta_{2j}\;*\;quadratic+r_{ij}.\\ Intercept & \beta_{0j}=\gamma_{00}+u_{0j}.\\ Linear & \beta_{1j}=\gamma_{10}+u_{0j}.\\ Quadratic & \beta_{2j}=\gamma_{20}+u_{2j}. \end{array}$$

**Table 3:** Linear and quadratic trends of perceived changes in personality for the Self and people in general.

	Self			People in general		
	Intercept	Linear	Quadratic	Intercept	Linear	Quadratic
Agreeableness	3.75	.12***	.01*	3.51	.21***	-.01*
Conscientiousness	3.96	.13***	-.02***	3.68	.26***	-.03***
Emotional stability	3.61	.19***	-.02***	3.38	.30***	-.03***
Openness to experience	4.04	-.06*	-.02***	3.63	-.34***	-.02***
Social vitality	3.65	-.10***	-.01***	3.79	-.28***	-.01***
Social dominance	3.79	.16***	-.02***	3.65	.07**	-.03***

*** *p* < .001.

** *p* < .01.

* *p<* .05.

The coefficients represent how closely participants’ ratings corresponded to the coefficients representing the trends. Note that a positive coefficient for the linear trend indicates that ratings increased over time. For the quadratic coefficient, a positive coefficient indicates an increasing then decreasing trend, whereas a negative coefficient represents a decreasing than an increasing trend.

The linear trend was significant for all traits for ratings of the self and others. As can be seen from the coefficients in Table 3, participants thought that emotional stability, agreeableness, conscientiousness, and social dominance would increase over time for themselves and for people in general. In contrast, participants thought that openness and social vitality would decrease over time for themselves and for people in general. Although the quadratic trends were also significant, with the exception of self-ratings of openness and ratings of social dominance for others, they were weaker than the linear trends (*p*s < .001), and so the linear trends represent the primary changes across time.

**Fig 1:**
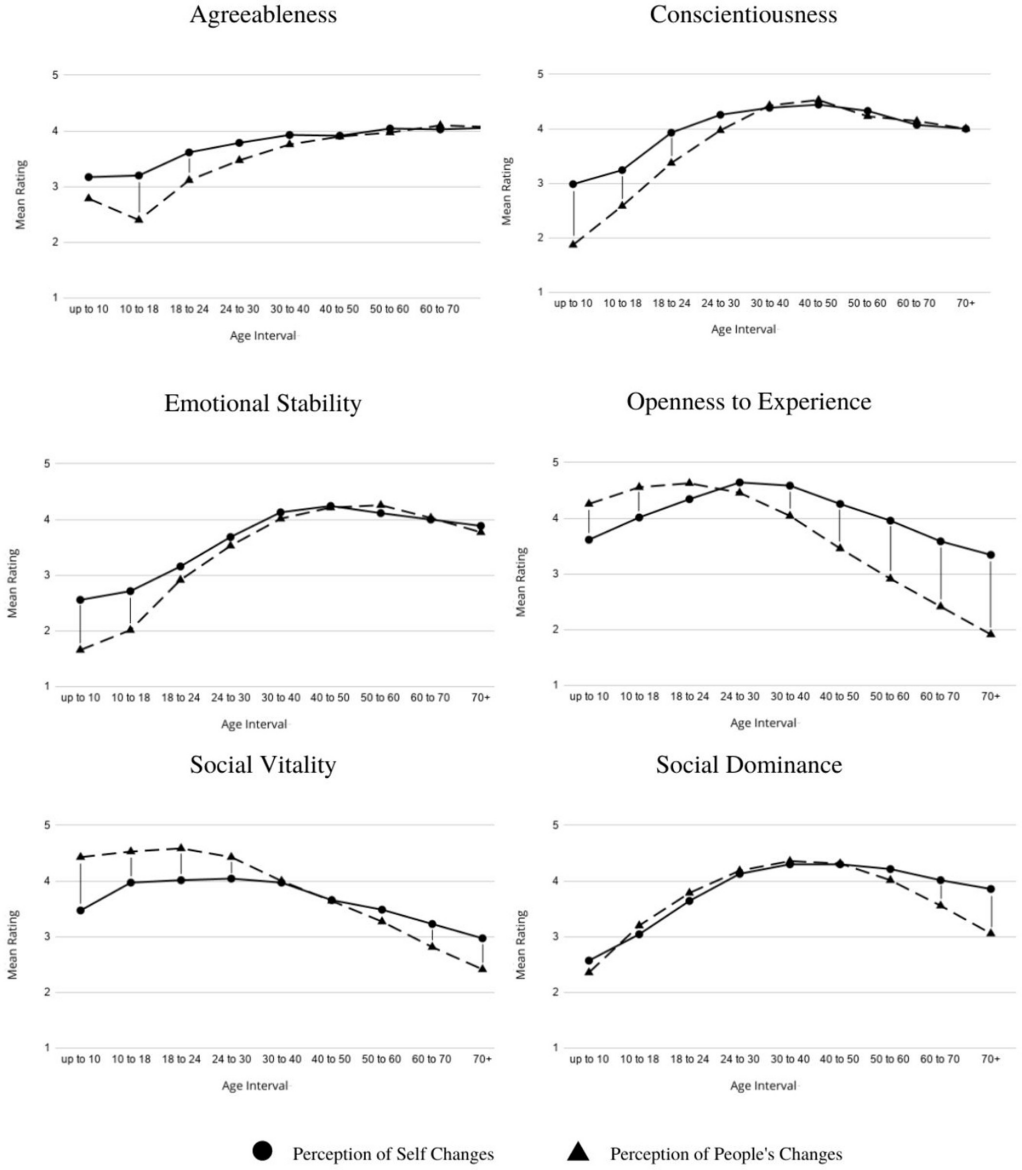
Mean ratings for the perception of changes of the self and the perception of people’s changes over the given age intervals across the lifespan for FFM trait.

To provide a context for understanding these results, charts representing perceived mean level changes for self-ratings and people-ratings are presented below (Fig 1). This figure also contains a summary of paired comparisons (the two ratings that were made at each interval) that were conducted as part of a series of 2 (self vs. people) by 9 (time interval) ANOVAs that are described below. The paired comparisons were done using tests with Sidak correction.

**Correspondence between perceived changes in personality across time and actual personality changes across time.** The changes we found in participants’ perceptions of changes over time in personality appeared to correspond to the changes that have been found in previous research on changes in personality over time [9,32]. To examine such correspondence, we conducted a series of multilevel models in which we regressed participants’ perceptions of change onto measures of change based on the meta-analysis of Roberts et al. [9].

Roberts et al. reported standardized mean differences. These were calculated by subtracting the trait score from time n-1 from a trait score at time n (e.g., age 10–18 minus age up to 10 years old) and dividing this mean difference by the standard deviation of the scores from time n-1 (the procedure was described inaccurately in the published paper, Roberts, personal communication). We calculated a difference score for each participant for each time period following the same procedure. There were seven difference scores. These scores allowed us to examine the correspondence between participants’ perceptions of change and the changes reported in the meta-analysis.

In these analyses, difference scores were nested within persons. Difference scores for each time period were regressed onto the difference score estimated by the meta-analysis for the corresponding time period. As before, each trait was analyzed separately, and separate analyses were done for self-ratings and ratings of others. The general model is presented below. In these models, “meta” is the standardized difference score. Meta was entered group-mean centered. The results of these analyses are summarized in Table 4.


$$\begin{array}{ll} Within‐person & y_{\mathrm{ij}}=\beta_{0j}+\beta_{1j}\;*\;meta+r_{\mathrm{ij}}.\\ Intercept & \beta_{0j}=\gamma_{00}+u_{0j}.\\ Meta & \beta_{1j}=\gamma_{10}+u_{0j}. \end{array}$$

**Table 4:** Correspondence between perceived changes in personality traits over the lifespan and changes estimated by meta-analysis.

	Self-ratings		People-ratings	
	Slope	*t*-ratio	Slope	*t*-ratio
Agreeableness	0.20	< 1	0.12	< 1
Conscientiousness	-0.54	1.95	0.45	< 1
Emotional stability	2.01	7.48**	3.24	11.33**
Openness to experience	1.15	4.90**	1.23	5.75**
Social vitality	0.89	3.03*	0.89	3.30*
Social dominance	1.36	5.74**	0.98	2.67*

* *p <* .01.

** *p <* .001.

The results of the analyses of the correspondence between the changes found in the meta-analysis and perceived changes in the self and the correspondence between the changes found in the meta-analysis and perceived changes in people in general were similar. We found significant positive relationships between participants’ perceptions of personality changes and the changes estimated by the meta-analysis of Roberts et al. in the analyses of openness, emotional stability, social vitality, and social dominance. There was no significant relationship between perception of changes and changes estimated by the meta-analysis for agreeableness and conscientiousness.

*Relationship between self-ratings across time and ratings for people across time for personality traits*. To examine the relationship between perceived personal changes in personality and perceived changes in the personality of others, we conducted a series of multilevel models in which we regressed participants’ perceptions of self onto perception of people in general (others in the model below). Intervals were nested within participants, and perceptions of others were entered group mean-centered. The model is presented below, and the results of these analyses are summarized in Table 5.


$$\begin{array}{ll} Within‐person & y_{\mathrm{ij}}=\beta_{0j}+\beta_{1j}\;*\;\mathrm{others}+r_{\mathrm{ij}}.\\ Intercept & \beta_{0j}=\gamma_{00}+u_{0j}.\\ Others & \beta_{1j}=\gamma_{10}+u_{0j}. \end{array}$$

**Table 5:** Relationship between Self-ratings and the ratings for people in general for personality traits over the lifespan.

	Coefficient	t-ratio	p-value
Agreeableness	0.26	4.64	< .001
Conscientiousness	0.42	8.20	< .001
Emotional Stability	0.56	10.73	< .001
Openness to experience	0.27	4.57	< .001
Social vitality	0.23	3.93	< .001
Social dominance	0.44	6.59	< .001

As can be seen from this summary presented in Table 5, the coefficients representing correspondence were all significant and all positive. This means that, to vary degrees, people thought others would change in the same ways they thought they themselves would change.

*Differences between self-ratings and ratings of people in general*: *Evaluating the primus inter pares (PIP) effect*. The previous analyses found that people believe that others will change as they do (more or less); however, these analyses did not directly test mean differences between ratings of the self and ratings of others. The last set of analyses did this and in so doing addressed the usefulness of the primus inter pares (PIP) effect as an explanation of perceived changes personality. These analyses were two (self vs. other) by nine (intervals) ANOVAS, with a decomposition of the intervals into trends. Given the prominence of the linear trend in the previous analyses of these ratings, we focused on the interaction of linear trend and the target of the ratings (self vs. others).

A summary of some of the results of these analyses is presented in Table 6. Consistent with the results of the multilevel modeling analyses, there were significant linear trends for all ratings. Moreover, all of these linear trends were qualified by interactions with self vs other ratings. Looking at the trends in Fig 1, it appears that there are three patterns. For agreeableness, conscientiousness, and emotional stability, self-ratings start higher than other-ratings, but there are eventually no differences between the two. For openness to experience and social vitality, self-ratings are initially lower than other-ratings, but self-ratings are higher than other rating-ratings later in the lifespan. Finally, for social dominance, self- and other-ratings were similar for much of the lifespan, but self-ratings were higher than other-ratings for older people.

**Table 6:** Means ratings for FFM traits at each age interval for the self and others with post hoc analyses for mean differences.

		Age intervals								
		up to 10	10–18	18–24	24–30	30–40	40–50	50–60	60–70	70+
Agreeableness	Self	3.17	3.20	3.61	3.79	3.93	3.91	4.04	4.03	4.06
	Others	2.79	2.40	3.11	3.47	3.76	3.90	3.97	4.10	4.07
	p-diff	.09	.0001	.0001	.05	.29	.94	.72	.75	.95
Conscientiousness	Self	2.99	3.24	3.93	4.26	4.39	4.44	4.33	4.07	4.00
	Others	1.87	2.59	3.37	3.97	4.43	4.53	4.23	4.15	4.00
	p-diff	.0001	.001	.001	.08	.75	.53	.49	.66	1.00
Emotional stability	Self	2.56	2.71	3.16	3.69	4.13	4.24	4.11	4.00	3.89
	Others	1.66	2.01	2.91	3.53	4.01	4.21	4.26	4.03	3.77
	p-diff	.0001	.0001	.15	.31	.45	.85	.40	.86	.57
Openness to experience	Self	3.61	4.01	4.34	4.64	4.59	4.26	3.96	3.59	3.34
	Others	4.26	4.56	4.63	4.46	4.04	3.46	2.91	2.41	1.91
	p-diff	.002	.001	.07	.22	.001	.0001	.0001	.0001	.0001
Social vitality	Self	3.47	3.97	4.01	4.04	3.97	3.66	3.49	3.23	2.97
	Others	4.43	4.53	4.59	4.43	4.00	3.64	3.27	2.81	2.41
	p-diff	.0001	.008	.002	.03	.86	.93	.22	.015	.003
Social dominance	Self	2.57	3.04	3.64	4.13	4.30	4.30	4.21	4.01	3.86
	Others	2.36	3.20	3.79	4.19	4.36	4.31	4.01	3.56	3.06
	p-diff	.32	.49	.49	.74	.72	.92	.20	.005	.0001

One way to understand these differences is to examine linear trends for self and other for each rating. Differences in the trends for self- and other-ratings were tested by the interaction term in the analyses described above. For agreeableness, conscientiousness, and emotional stability, the linear trends for self-ratings were less positive than the linear trends for other-ratings, agreeableness, 7.01 vs 12.39; conscientiousness, 7.53 vs. 15.46; emotional stability, 11.64 vs. 17.87. For openness and social vitality, the linear trends for self-ratings were not as negative as the linear trends for other-ratings, openness, -3.53 vs. -20.23; social vitality, -5.67 vs. -16.61. For social dominance, the linear trend for self-ratings was stronger than the linear trend for other-ratings (9.37 vs. 4.46).

## Discussion

In the present paper we propose a new mechanism that can explain (at least in part) peoples’ beliefs about how their personalities will change over the course of their lives. We assumed that (1) when thinking about how their personalities will change over their lives, people refer to normative based beliefs about how personality changes over the lifespan, and these normative based beliefs parallel actual changes in personality over the life course, and (2) although people’s expectations are influenced by normative beliefs, people may self-enhance by perceiving themselves to be superior to others in terms of how much they will change compared to others. Regarding this, we followed Codol’s [7] concept of superior conformity of the self.

In two studies, we examined people’s beliefs about how their personalities change over the lifespan in terms of the traits that constitute the FFM of personality. The results confirmed our expectations that how people thought they would change over the life course would correspond to their beliefs about how others would change (normative-based beliefs). For all FFM traits, the relationships between these two ratings were positive and significant. Also, patterns of perceived changes over the life course for both the self and for others paralleled the pattern of actual changes as found in the meta-analysis of Roberts et al. [9].

The pattern of relationships between perceived changes and actual changes was similar for perceptions of the self and for perceptions of others. Specifically, significant positive relationships between perceived changes in personality and changes estimated by the meta-analysis of Roberts et al. were found for openness, emotional stability, social vitality, and social dominance. This normative pattern is such that individuals tend to become more functionally mature over the life course, but they tend to decrease on dimensions that include energy and activity (e.g., extraversion) [8,33].

We did not find that significant relationships between changes estimated by the meta-analysis and perceived changes in the self for agreeableness and conscientiousness. Although relationships based on the standardized mean differences (i.e., the method used in the meta-analysis) were not significant, for both self-perceived agreeableness and conscientiousness the trend analyses paralleled the normative pattern of personality development, i.e., increase over time. One possible explanation for the discrepancy between the conclusions based on these two types of analyses is that over time people thought that they were becoming more agreeable and conscientious (as is the norm), the age intervals during which these changes were presumed to occur did not correspond to the changes found in the meta-analysis. For example, we found gradual, consistent increases in self-perceived agreeableness, whereas the meta-analysis found only one significant increase, from 50 to 60. Clearly, understanding such discrepancies requires further study.

The trend analyses found that the primary change across time for all traits for ratings of both the self and others was a linear trend. Participants expected that emotional stability, agreeableness, conscientiousness, and social dominance would increase over time, whereas they expected that openness and social vitality would decrease. These results are consistent with analyses of age-related differences in personality that suggest that measures of agreeableness and conscientiousness are positively correlated with age, whereas measures of extraversion and openness are negatively correlated with age [14].

The present results are also consistent with the previous findings that have indicated that expectations for personality traits in age-graded roles correspond to the actual pattern of personality development across the life course [8]. Wood and Roberts [8] argued that general age expectations are to great extent a function of the beliefs about age-graded roles such as being a student, a parent, or an employee. These baseline beliefs are culturally shared and can influence people’s expectations about how everyone should behave at different age and age-graded roles. This can be a part of the normative social mechanisms that influence the personality development.

Previous research has focused on expectations for changes in the personalities of others. The results of the current study contribute to the existing literature by demonstrating that perceptions of normative personality changes over the life course correspond to the expectations people have about how their own personalities will change. This suggests that people internalize expectations for normative personality changes.

Our results support the proposal of Wood & Roberts [8] that “the expectations of how people behave at different ages or in age-graded roles act as standards that direct the observed patterns of mean-level personality development” (p. 1494). The normative social mechanism associated with social expectations about how people should be at different ages and age-graded roles can be powerful influences on personality development when these expectations are internalized and become expectations for personal changes. The results of the present research suggest this can be the case.

### Perceived changes in personality as an illustration of being first among equals

Although the correspondence between self- and other-rating suggest that participants expected to change over the life span asthey expected others to change, they also tended to see themselves as better than others in some ways. The general pattern of results can be summarized as follows: for traits associated with maturation (agreeableness, conscientiousness, and emotional stability) participants believed they started better than others (self-ratings were higher than others-ratings early in the lifespan), whereas for traits that generally decline with age (openness to experience and both facets of extraversion, social vitality and social dominance), participants expected they would age better than others (self-ratings were higher than other-ratings later in the lifespan). Participants believed that although they mature and age similar to others, they matured earlier than others, and they expected to decline less over time than other people decline.

In a classic paper, describing the results of 20 studies, Codol [7] proposed a mechanism that could reconcile two competing motives in self-evaluation, the motive to be similar to everyone else (to be a member of a collective) and the motive to be better than everyone else. Codol demonstrated that people tend to think they possess the same characteristics as members of their groups, but they possess these characteristics more strongly. In essence, people tend to think that they are “super members” of their groups. If being hard-working is part of a group’s identity, they are more hard-working than the typical member of the group. If being relaxed is part of a group’s identity, they are more relaxed than the typical member of the group.

Codol’s proposed mechanism, in combination with normative based beliefs, can account for many of the differences between self- and other-ratings we found. These differences can be characterized by one of two broad patterns. For traits associated with maturation (agreeableness, conscientiousness, and emotional stability) participants believed they were more mature than others early in life (self-ratings were higher than other-ratings for intervals early in the lifespan), and these differences tended to diminish over time. Such a pattern represents a combination of Codol’s mechanism (more mature than others at the outset) and normative-based beliefs (eventually, most people mature).

In contrast, for traits that generally become weaker with age (openness to experience and both facets of extraversion, social vitality and social dominance), participants expected that, similar to others, these traits would weaken for them. This reflects the influence of normative based beliefs. Simultaneously, participants also believed that the decline in the strength of these traits would be weaker for them that it would be for others. Self-ratings for these traits later in the lifespan were higher than other-ratings later in the lifespan. This reflects the influence of Codol’s mechanism. Participants believed they would decline (they would be among equals), but they thought their declines would be weaker than others’ (they would be first among equals).

### Limitations and future directions

Similar to any study, the generalizability of the present results is limited by the sample we examined. The correspondence between the perceptions of our participants and the results of the meta-analysis, which included 92 samples, suggests that our sample did not differ in ways that that were relevant to the mechanisms we were studying. Nevertheless, it is possible that our sample differed from the samples studied in previous research in ways that were related to the mechanisms we were studying.

Putting aside concerns about the sample, there is also the issue of the domain we examined. We chose the FFM as a context to study this topic because there was an existing body of research that had examined how people in fact changed across the life span. Nevertheless, we cannot generalize from our findings about FFM traits to other characteristics. For example, how might people change in terms of the Big Two, agency and communion [34]?

There is also the issue of individual differences in the differences people see between changes in themselves and changes in others. Although to our knowledge, such individual differences have not been studied per se, existing research on the self-enhancement bias suggests that differences between perceptions of changes in the self and in others may be related to well-being [35]. Such relationships could concern differences in mean levels–the extent to which people see themselves as more conscientious than others could be positively related to well-being. They could also concern differences in trajectories. For a positive trait that is perceived to increase for self and for others, differences between how much people think they will increase vs how much they think others will increase may be positively related to well-being. Unfortunately, we did collect the data needed to examine such possibilities.

Regardless of these concerns, we think the present study provides insights into how people think they will change over the course of their lives. Moreover, we believe that the present results suggest that more attention needs to be paid to Codol’s body of work. We believe that his work has not received the attention it should receive in studies of temporal comparison.
